# Outcomes of Modified Tissue Selection Therapy Stapler in the Treatment of Prolapsing Hemorrhoids

**DOI:** 10.3389/fsurg.2022.838742

**Published:** 2022-03-03

**Authors:** Chenchen Yuan, Chongjun Zhou, Rong Xue, Xiaofeng Jin, Chun Jin, Chenguo Zheng

**Affiliations:** ^1^The Second Affiliated Hospital and Yuying Children's Hospital of Wenzhou Medical University, Wenzhou, China; ^2^Department of Graduate Education Management Division, Wenzhou Medical University, Wenzhou, China

**Keywords:** tissue selection therapy stapler, prolapsing hemorrhoids, modified, complication rate, conformal

## Abstract

**Objective:**

Tissue selection therapy staplers (TSTs) are widely used to treat prolapsing hemorrhoids; however, some disadvantages exist. We describe a modified technique for the treatment of prolapsing hemorrhoids, with the aim of minimizing the risk of anal stenosis and anal incontinence and reducing the impact of postoperative complications from the stapling technique. We applied a modified TST procedure, and the preliminary data were used to test the efficacy and safety of this new technique.

**Methods:**

We conducted a retrospective study of patients who underwent modified TST for prolapsing hemorrhoids at our department between January 2018 and January 2020. All patients received a modified TST. Most prolapsing hemorrhoids were not segmentally resected and were instead selectively removed. The demographics, preoperative characteristics, postoperative complications, therapeutic effects, and patient satisfaction were collected and analyzed.

**Results:**

A total of 106 patients were included in the study; 53 were men and 53 women (mean age, 49.24 years). The mean operative time was 55.01 min, and the mean hospital stay was 7.82 days. After surgery, three patients experienced bleeding (2.83%), 2 patients experienced anal discharge (1.89%), 2 patients experienced tenesmus (1.89%), and 5 patients experienced anal tags (4.72%). Anal incontinence, persistent post stapler pain, rectovaginal fistula and anal stenosis did not occur. Two patients developed recurrent symptomatic hemorrhoids (1.89%). The total effective rate of the surgery and the total satisfaction rate of the patients was 97.17%.

**Conclusions:**

The modified tissue selection therapy stapler technique was a satisfactory and economical treatment for prolapsing hemorrhoids at a follow-up period of 1 year. The modified TST was associated with reduced anal stenosis and anal incontinence, less persistent post stapler pain and a minimal risk of rectovaginal fistula.

## Introduction

Prolapsed hemorrhoids are a common anorectal disease, and their incidence has been reported to be ~50.1% among adults (1). Surgery is the most effective treatment, especially for severe prolapsing hemorrhoids (2, 3). Milligan-Morgan hemorrhoidectomy (MMH) is the gold standard for resecting hemorrhoids. Although it has been widely used in clinical practice, there might be disadvantages, such as slow healing of the wounds, a poor suspension effect after mucosal resection, residual hemorrhoids, and severe postoperative pain. A procedure for treating prolapse and hemorrhoids (PPH) was invented by Longo (4) to treat circumferential mixed hemorrhoid patients, and the aim of the Longo technique is to promote the reduction of the anal cushion by resecting the submucosal tissue of the lower rectum and anastomosing the broken end of the mucosa. However, negative effects of PPH have been reported, and the recurrence rate of prolapsed hemorrhoids is high (5, 6). Postoperative complications and adverse events have been reported, including acute urinary retention (7), chronic sustained pain (8), anastomotic stenosis (9), and anal incontinence (10).

The tissue selecting technique (TST) is a new minimally invasive technique for prolapsed hemorrhoids. It maintains a normal mucosa bridge while simultaneously reducing surgical trauma, and it has achieved desirable efficacy after PPH. However, its side effects have been reported in recent years, such as pendant expansion and anastomotic bleeding (11). On the other hand, single-window anoscopy, double-window anoscopy and triple-window anoscopy were found to be inaccurate for the resection of hemorrhoids with variable shapes and sizes, and the cost of the TST device is very high (470–627 dollars).

Thus, we developed a novel method based on the TST approach that we are calling modified TST to overcome the limitations of TST. The hemorrhoids were conformally and selectively removed according to their size and quantity, and the relatively normal anal pads were preserved to maintain the physiological function of the anus with the goal of minimizing the risk of anal stenosis and anal incontinence and reducing the impact of postoperative complications of the stapling technique. This clinical retrospective analysis was performed to observe and analyze the efficacy and complications associated with modified TST for prolapsing hemorrhoids.

## Materials and Methods

### Participants

From January 2018 to January 2020, a total of 106 patients underwent modified TST at the Second Affiliated Hospital and Yuying Children's Hospital of Wenzhou Medical University. The inclusion criteria were patients who were aged >18 y and <75 y, had grade III–IV mixed prolapsing hemorrhoids according to the Goligher classification (12), had more than four consecutive o'clock sites of circumferential hemorrhoids, and planned to receive modified TST. The exclusion criteria were patients with severe primary diseases of the cardiovascular system, those who had other colorectal disorders and dysfunctions (e.g., tumor and inflammatory bowel disease), and those who had previously undergone surgery for mixed prolapsing hemorrhoids (traditional or stapled). The studies involving human participants were reviewed and approved by the Ethics Committee of the Second Affiliated Hospital and Yuying Children's Hospital of Wenzhou Medical University. The participants provided written informed consent to participate in this study.

### Data Collection

All data maintained in the computer database after the surgery were collected, retrospectively. The following parameters were recorded and analyzed: clinicopathological characteristics, including age, sex, body mass index (BMI), presenting symptoms, surgical duration, intraoperative blood loss, hospital stay, and hospital costs. Postoperative immediate complications, including the Numerical Rating Scale (13) and the additional use of analgesics, were collected. Postoperative digital anal and anoscopy examinations were conducted at our outpatient department every week until full recovery. Telephone follow-up was conducted every 3 months after surgery until 1 year. Patients were invited to the outpatient clinic for a final evaluation if any severe complications appeared during the follow-up period. Patient satisfaction and long-term complications (including anastomotic bleeding, persistent post stapler pain, anal stenosis, anal incontinence, anal discharge, anal tags, tenesmus, rectovaginal fistula and recurrence) were also recorded during the follow-up period.

### Surgical Procedures

**Figure 1:**
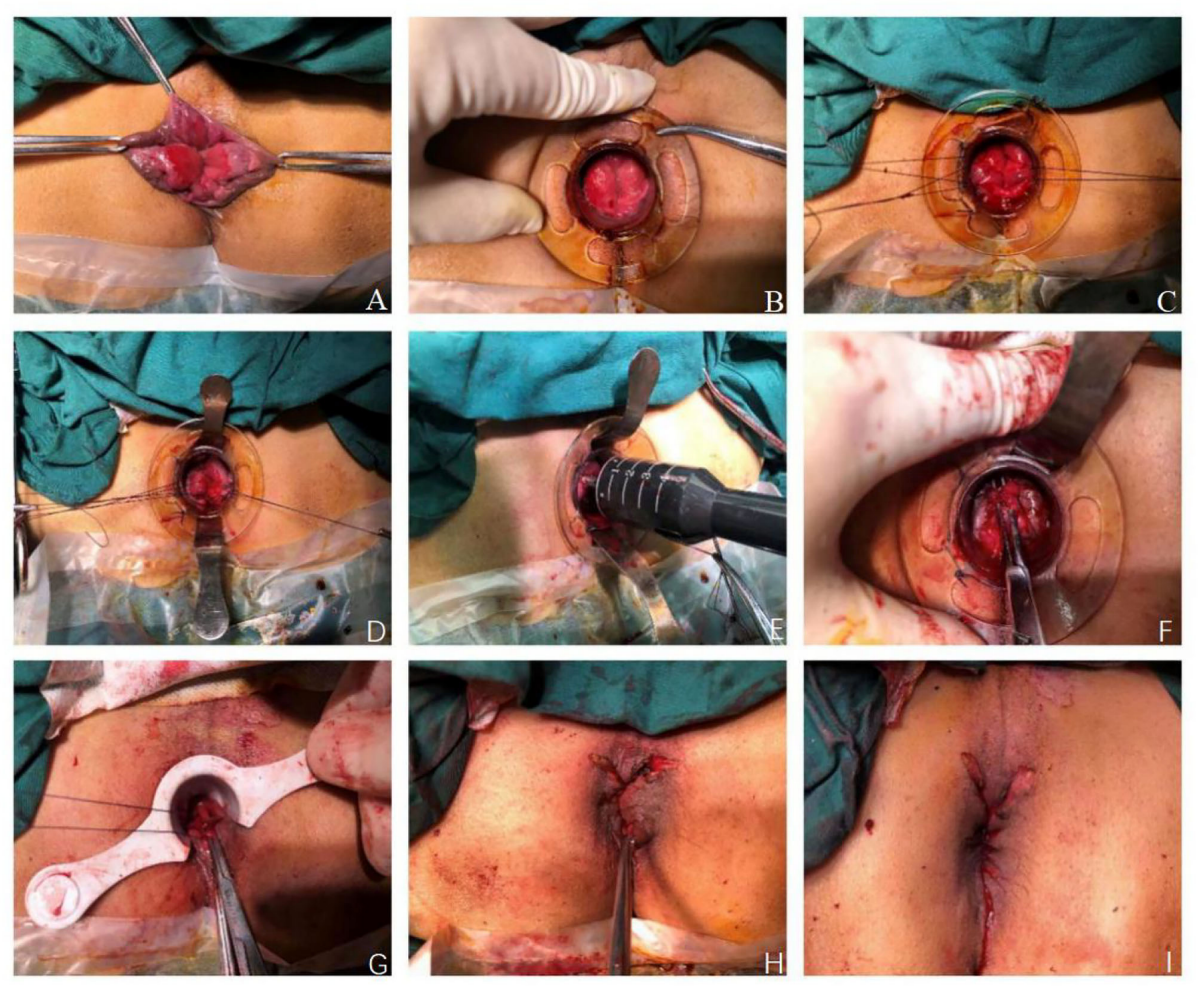
Patients with prolapsing hemorrhoids treated with modified TST. **(A)** Exposing hemorrhoids with allis forceps. **(B)** An anoscope was inserted into the anus. **(C)** Purse string suture were made with 2-0 absorbable suture. **(D)** Two metal baffles were inserted. **(E)** Fired the stapler. **(F)** The bridges were separated. **(G)** The free ends of dissected mucosal bridges were ligated. **(H)** The external hemorrhoids were excised. **(I)** The perianal skin was repaired with absorbable sutures.

**Figure 2:**
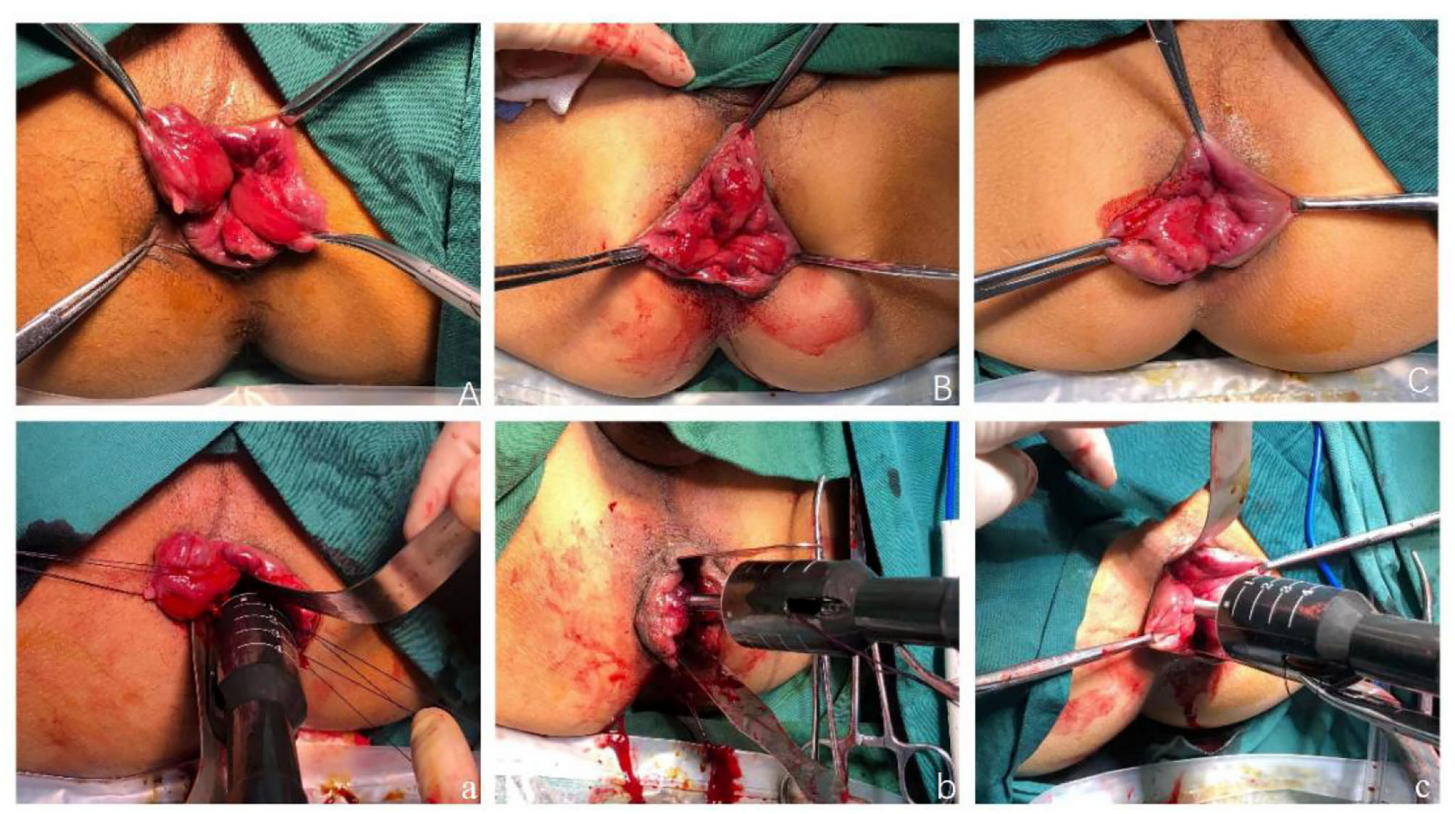
**(A)** The left anterior and right posterior mucosal membranes were retained. The preoperative pictures of the patients in (a). **(B)** The anterior and posterior mucosal membranes were retained. The preoperative pictures of the patients in (b). **(C)** The right mucosa membrane was retained. The preoperative pictures of the patients in (c).

**Figure 3:**
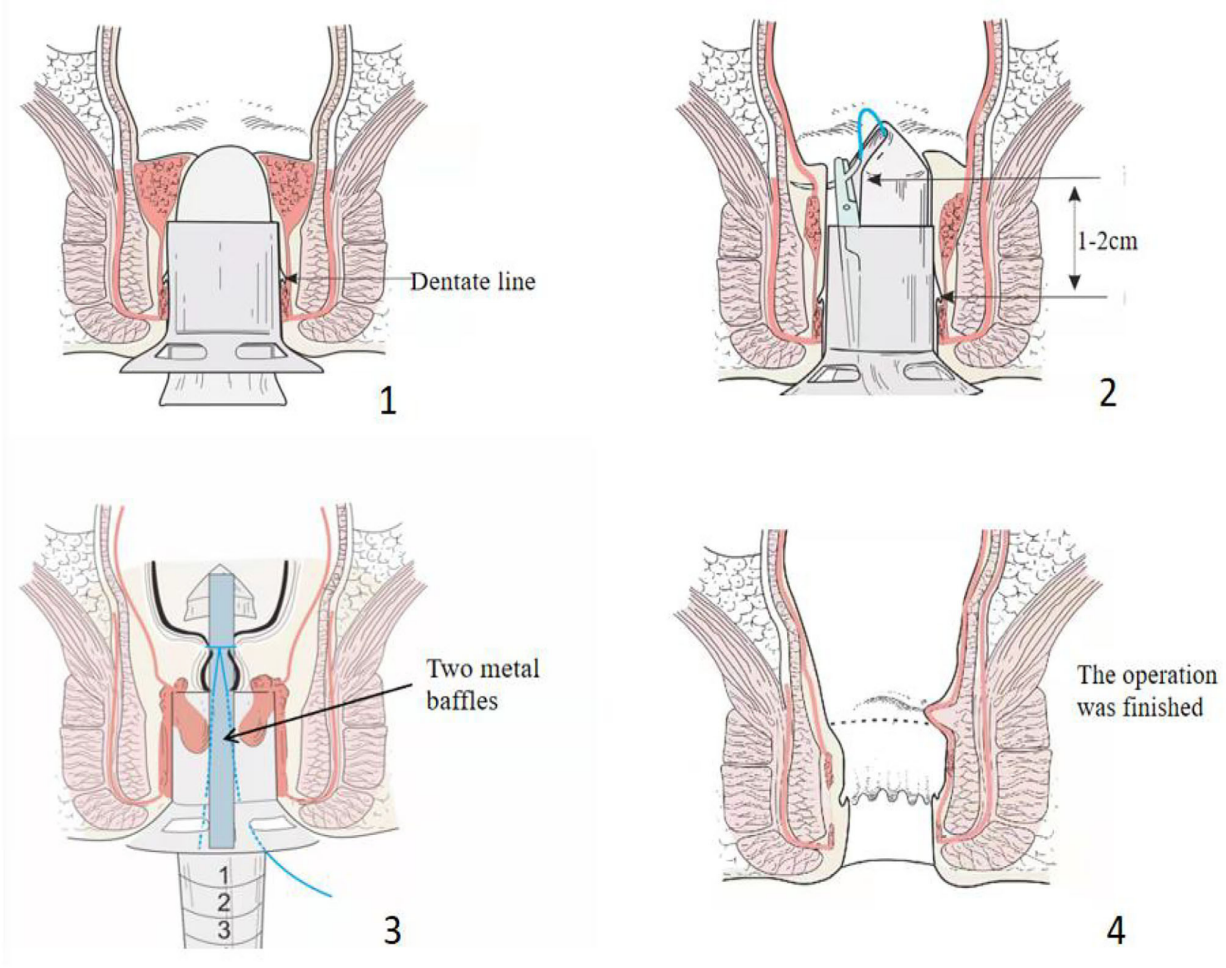
The modified technique shown in drawings to allow for a correct reproduction of the technique.

Modified TST was performed using the following steps: (1) The patient was placed in the lithotomy position after general anesthesia, exposing the hemorrhoids with allis forceps, observing the distribution of the hemorrhoids and choosing the mucous membrane that needed to be maintained (Figure 1A). (2) An anoscope (YI LIAN) was inserted into the anus at a position where the upper half of the hemorrhoids was exposed (Figure 1B). (3) Purse string sutures were made on the mucosa and submucosa 1–2 cm from the dentate line. If the hemorrhoids were large, we performed double purse ring sutures (Figure 1C). (4) Two metal baffles were used to preserve the relatively normal mucosa in any direction. The anterior and posterior mucous membranes are shown in Figure 1D. The hemorrhoids were conformally and selectively removed according to the size and quantity of the hemorrhoids as shown in Figure 2. Figures 2A–C correspond to the preoperative pictures of the patients in Figures 2a–c, respectively. The left anterior and right posterior mucosal membranes are shown in Figure 2a. The anterior and posterior mucosal membranes are shown in Figure 2b. The right mucosa membrane shown in Figure 2c was retained. (5) The purse strings were tied to the stapler, and then the stapler was fired (Figure 1E). These bridges were separated, and the free ends of the dissected mucosal bridges were separately ligated (Figures 1F,G). (7) The external hemorrhoids were excised appropriately, and finally, the perianal skin was repaired with absorbable sutures (Figures 1H,I). We have provided drawings (Figure 3) and a video to help surgeons understand and achieve full reproduction of these procedures.

### Postoperative Management and Follow-Up

Postoperative treatment consisted of standard nursing care and a semifluid diet. Patients experiencing postoperative pain within 1–3 days after the operation routinely received non-steroidal anti-inflammatory drugs (NSAIDs) twice a day as an analgesic. Patients were injected with additional opiates if necessary due to unbearable pain after routine analgesia.

Short-term postoperative complications were recorded during hospitalization: the frequency of additional injected postoperative analgesics were counted (0 indicating no unbearable pain and 1 indicating unbearable pain), and the patients' postoperative pain was recorded at seven time points after the operation (Day 1, Day 2, Day 3, Day 4, Day 5, Day 6, and Day 7), as assessed by the NRS score [0 indicating no pain and 10 indicating the worst pain (14, 15)].

A postoperative review was conducted at our outpatient department, and if the following symptoms appeared they were recorded: anastomotic bleeding (anastomotic hemorrhage found by anal examination, surgical intervention with 3#0 absorbable sutures were used for ligation and hemostasis); persistent post stapler pain as evaluated by NRS; anal stenosis [a condition in which the patients have difficulty in defecation and incomplete evacuation with a narrow stools caliber (16)]; anal incontinence [a lack of control over defecation, resulting in involuntary leakage of solid and/or liquid stool, with and without unintentional release of gas (17)]; anal discharge [perianal dampness or anal mucus secretion caused by the scar left by the surgery (18)]; anal tag [a perianal mass was pliable with an obvious foreign body sensation (11)]; tenesmus [the patient had a chief complaint of a sensation of rectal tenesmus (19)]; rectovaginal fistula [an opening allowing the passage of flatus and stool through the vagina (20)]; postoperative recurrence [continuous prolapse of perianal piles that recurred after hemorrhoidectomy (21)].

The efficacy was assessed 12 months after the operation, and the evaluation criteria (18) were defined as follows: marked effectiveness: the prolapse symptoms almost entirely disappeared; effectiveness: <50% prolapse symptoms remained compared with preoperative; ineffectiveness: >50% prolapse symptoms remained compared with preoperative. A patient satisfaction score (22) was obtained at 12 months by telephone follow-up. The scores ranged from 1 to 3, with 1 being satisfied with the outcome and 3 dissatisfied.

### Statistical Analysis

The data were statistically analyzed. Normally distributed continuous variables are expressed as the means and standard deviation (SD), and non-normally distributed continuous variables are presented as the medians and interquartile range (IQR). Categorical variables are shown as numbers and percentages. All data were analyzed with SPSS statistical version 25.0.

## Results

### Patient Characteristics and Clinical Data

**Figure 4:**
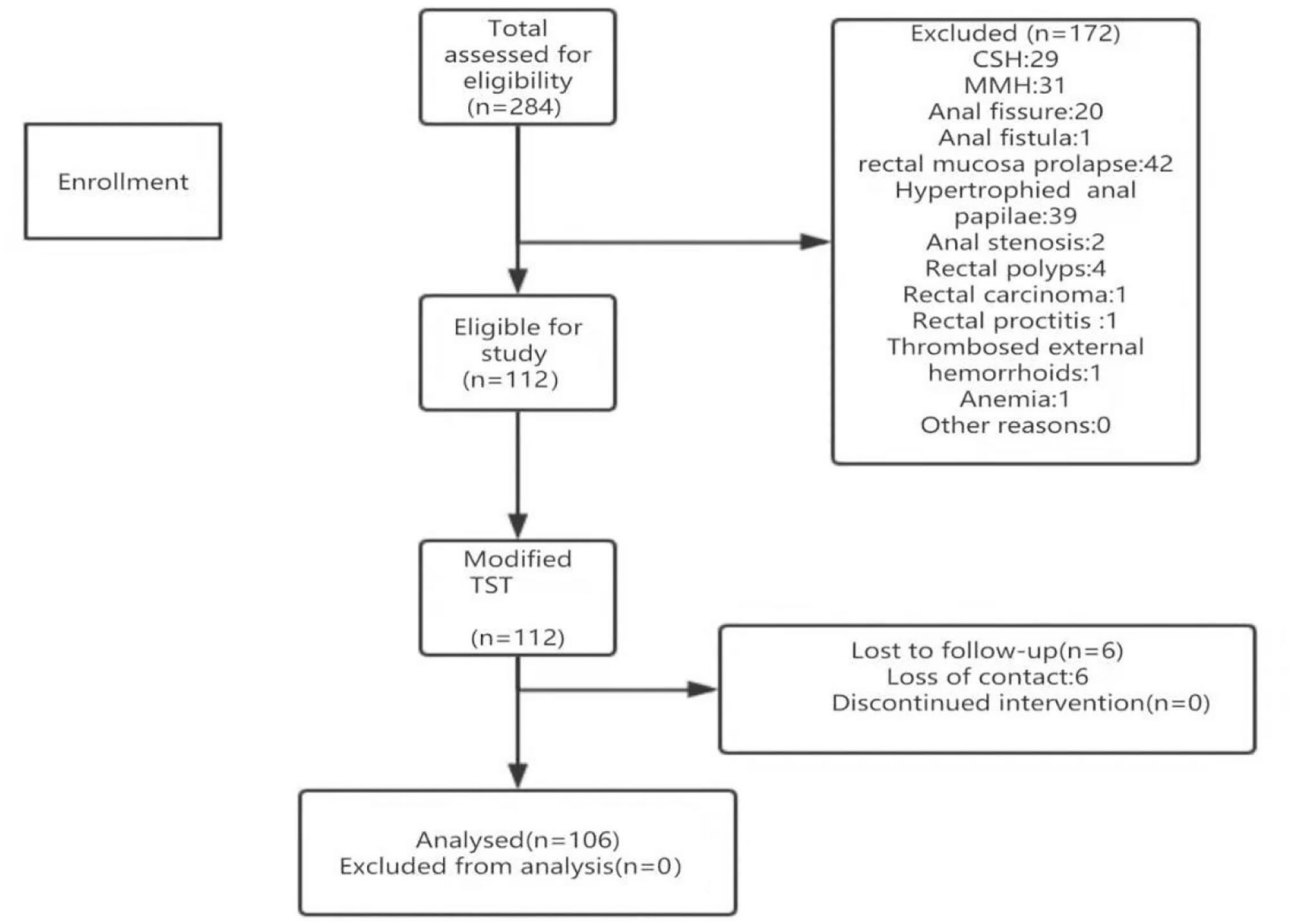
Participant enrollment and follow-up. CSH, circumferential stapled hemorrhoidopexy; MMH, Milligan-Morgan hemorrhoidectomy; TST, Tissue selecting technique.

A total of 112 eligible patients with grade III-IV prolapsing hemorrhoids were enrolled during the study period, with 106 undergoing modified TST (Figure 4). The follow-up period was 12 months. Table 1 shows the analysis of the demographic characteristics and the clinical data of the patients. Their mean age was 49.24 y (range, 18–75 y), and there were 53 men and 53 women. Their mean body mass index (BMI) was 23.46 kg/m^2^. The presenting symptoms of most patients were hematochezia and/or prolapse of hemorrhoids (99/106). The median operative time was 55.01 min (range, 25–95 min). The median intraoperative blood loss was 5 ml (range, 2–50 ml). The median hospitalization stay and hospitalization expenses were 7.82 d (range, 5–17 d) and 1938.95 dollars (range, 1415.93–3541.69 dollars), respectively.

**Table 1:** Patient demographics and clinical characteristics.

**Factors**	**Total (*n* = 106)**
**Age, mean, (SD), y**	49.24 (11.53)
**Sex**, ***n*** **(%)**	
Female	53 (50.00)
Male	53 (50.00)
**BMI, mean, (SD), kg/m** ^**2**^	23.46 (2.94)
**Presenting symptoms**, ***n*** **(%)**	
Hematochezia	4 (5.64)
Prolapse of hemorrhoids	12 (16.90)
Both of the above	83 (78.30)
Others	7 (9.86)
**Intraoperative blood loss, median (IQR), ml**	5 (7)
**Operative time (SD), sec**	55.01 (14.50)
**Hospitalization stay, mean (SD), d**	7.82 (2.38)
**Hospitalization expenses, median (IQR), dollars**	1938.95 (381.44)

*BMI, Body mass index; SD, standard deviation; IQR, Interquartile range*.

### Patient Satisfaction and Overall Efficacy

The majority of patients were satisfied with their surgery. Overall, 97.17% (103/106) of the patients reported being satisfied or partially satisfied by resolution of their troubling symptoms (score <3) after the procedures. The total effective rate was achieved in 97.17% (103/106) of patients after modified TST. The total satisfaction rate and total effective rate of the patients are presented in Table 2.

**Table 2:** Efficacy assessment and patient satisfaction.

**Results**	**Total (*n* = 106)**
**Efficacy assessment**, ***n*** **(%)**	
Markedly effectiveness	92 (86.79)
Effectiveness	11 (10.38)
Ineffectiveness	3 (2.83)
**Patients' satisfaction**, ***n*** **(%)**	
Satisfied	97 (91.51)
Partially satisfied	6 (5.66)
Dissatisfied	3 (2.83)

*The total effective rate = markedly effectiveness rate + effectiveness rate. The total satisfaction rate = satisfied rate + partially satisfied rate*.

### Complications

**Figure 5:**
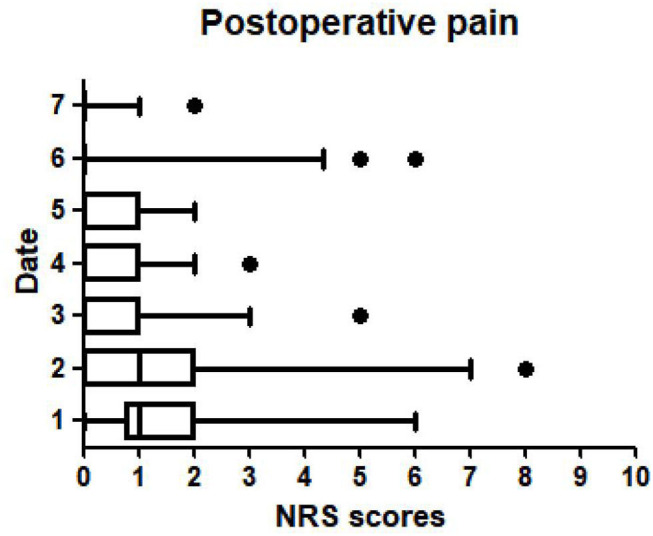
Postoperative pain was recorded by the NRS scores from the first day to the seventh day after the operation.

Short-term postoperative complications, especially postoperative pain, were recorded by the NRS scores from the first day to the seventh day after the operation (Figure 5). The second day after the operation had the highest score, representing some patients experiencing intractable pain. The pain score was the lowest on the seventh day, indicating that the postoperative pain was relieved. Eight patients received an additional dose of analgesics (7.54%). Other complications were analyzed in detail (Table 3). The telephone follow-up and/or outpatient follow-up showed that no participants had anal incontinence, persistent post stapler pain, rectovaginal fistula (RVF) or anal stenosis. All complications were recorded, and the incidence of anal tags (4.72%) was the highest, followed by anastomotic bleeding (2.83%) (Table 3). Two of the 106 patients (1.89%) had symptomatic anal discharge and tenesmus. Two patients developed recurrent symptomatic hemorrhoids, leading to a yearly recurrence rate of hemorrhoids of 1.89% (2/106) (Table 3).

**Table 3:** Complications, *n*.

**Complications**	**Total (*n* = 106)**
**Short-term complications**	
The frequency of additional injected postoperative analgesics, *n* (%)	8 (7.54)
Postoperative pain, median (IQR)	1 (1)
**Long -term complications**, ***n*** **(%)**	
Anastomotic bleeding	3 (2.83%)
Persistent post stapler pain	0
Anal stenosis	0
Anal incontinence	0
Anal discharge	2 (1.89%)
Anal tag	5 (4.72%)
Tenesmus	2 (1.89%)
Rectovaginal fistula	0
The 1-year recurrence rate	2 (1.89%)

*SD, standard deviation; IQR, Interquartile range*.

## Discussion

The experience of clinical observation and medical research indicates that surgical resection combined with other therapeutic methods should be given priority for the treatment of grade III–IV mixed prolapsing hemorrhoids, although debate exists as to which of the multitudinous surgical procedures is the most clinically effective (23). Pata et al. (24) described the two directions of surgical treatments for hemorrhoids in the first 20 years of the 2000s: modified traditional techniques and minimally invasive techniques. Based on the concept of minimally invasive surgery and reducing surgical trauma, this study introduced a modified procedure for the surgical management of hemorrhoidal disease. The surgeon innovatively used metal plates to readjust the surgical scope on the basis of the individual patient's clinical condition. The hemorrhoids were conformally selectively removed according to their size and quantity, and the relatively normal anal pads were preserved to maintain the physiological function of the anus.

The present study demonstrated that the modified TST technique achieved a superior effect compared to that of traditional TST in the management of prolapsing hemorrhoids. Lin et al. (21) reported that the 1-year recurrence rate of prolapsing hemorrhoids after traditional TST was 3.3%. It was speculated that this high recurrence rate might be associated with the patients not distinguishing between remnant prolapsed piles and anal tags from a recurrent prolapse (25). The patients therefore underwent a specialist examination to evaluate the actual cause of their recurrent symptoms. Ortiz et al. (26) also reported that the incidence of recurrent prolapsing hemorrhoids after PPH was as high as 25.9%. It was assumed that modified TST might decrease the recurrence rate. The recurrence rate of symptomatic hemorrhoids after MMH procedures were slightly higher (1.89% for modified TST vs. 2.6–2.7% for MMH) with a follow-up of 1 year, as shown in Table 4. The overall procedural complication rates of stapled hemorrhoidectomy ranged from 2 to 68% (29). We therefore believe that modified TST, compared with traditional TST, PPH, and MMH, conformally selectively excises the hemorrhoidal-bearing area, leading to a possible reduction in the recurrence rate. However, a few authors (34) have reported that stapled hemorrhoidopexy during the surgical procedures excluded a direct correlation with an increased rate of complications. The limitations of our study included the fact that it had a small sample size, lacked a control group, and was a retrospective study with a discrete sample number. Further investigation with a larger sample size, long-term postoperative follow-up and multicenter prospective studies is necessary to clarify this point.

**Table 4:** The incidence of postoperative complications of different surgical procedures.

**Complications**	**Modified TST**	**TST**	**PPH**	**MMH**
Anastomotic bleeding	2.85%	2.5% (22)	1–11% (7, 19)	1–2.6% (27, 28)
Persistent post stapler pain	0	0.9% (18)	1.4–8% (7, 19)	0–5.4% (18, 19)
Anal stenosis	0	0(9, 22)	0.2–7.5% (7, 9, 27, 29, 30)	2.6% (27)
Anal incontinence	0	1.4% (18)	3.2–31% (7, 19, 31)	7.2% (18)
Anal discharge	1.89%	1.4% (18)	38% (31)	10.4% (18)
Anal tag	4.72%	8.6% (11)	1.8–80% (27, 30, 32, 33)	3.7–21% (27, 33)
Tenesmus	1.89%	NR	14% (19)	8% (19)
Rectovaginal fistula	0	0(18, 21, 22)	0.2% (19)	0(18, 21)
The 1-year recurrence rate	1.89%	3.3% (21)	4.6–25.9% (7, 19, 26, 27, 32)	2.6–2.7% (21, 27)

*NR, not reported; PPH, procedure for prolapse and hemorrhoids; MMH, Milligan-Morgan hemorrhoidectomy; TST, tissue selecting technique*.

It has been shown that surgical techniques and postoperative analgesics are associated with acute and chronic pain (35). This study found that the NRS scores of postoperative pain were low, and they were strongly correlated with the use of postoperative analgesics. This study routinely used non-steroidal anti-inflammatory drugs (NSAIDs) for analgesia twice a day to alleviate acute pain after the operation. The modified TST places the staple line 1 cm from the dentate line where there are fewer sensory nerves and far from the sensitive epithelium of the anal canal, which helped to relieve pain and prevent edema. The causes of postoperative pain in patients after stapled techniques have been reported to be a purse string suture placed deep and close to the levators, resulting in low-grade inflammation along with continuous stimulation, especially during the first postoperative defecation (30). A consensus statement (29) summarized the importance of comprehensive knowledge of the local anatomy and a proper choice of surgical techniques.

Residual anal tags were found after PPH in 1.8–80% of patients as reported in the literature, for which the incidence of residual anal tags was the highest among the various procedures (4.72% for modified TST vs. 8.6% for traditional TST vs. 3.7–21% for MMH) (30, 32, 36). The modified TST preferentially removes the remaining circumferential external hemorrhoids and asymptomatic skin tags, which are considered to be sources of anal discomfort and itching. The present study showed that there was no significant difference in persistent post stapler pain between the modified TST and the other surgical procedures. This may be associated with retaining an appropriate mucosal bridge and full drainage to reduce postoperative anal edema.

The current study showed that anal incontinence was not encountered in any patients treated with modified TST, while the incidence of postoperative anal incontinence after MMH may be as high as 7.2% (18). It was speculated that modified TST retained the non-pathologic anal cushions without affecting anal function and maintained the continence function of the rectum and the anus, thereby avoiding fecal incontinence and urgency to a large extent (30). Sturiale et al. (10) found that stapled hemorrhoidopexy was associated with a high incontinence rate. This was found to be related to the unsuitable low position of the staples or possibly the excessive inflammatory response around the staple line after the operation. Mascagni et al. (37) suggested that defecatory urgency or gas/fecal incontinence may be caused by excessive resection. Therefore, maximal preservation of the normal mucosa and the anal sphincter is able to alleviate anal continence and urgency and increase defecation control (38, 39).

The incidence of anal stenosis after PPH and MMH reported in the literature is 0.2–7.5 and 2.6%, respectively (27, 29). The patients in the PPH group had higher rates of anastomotic stricture cases (40) and a higher incidence of fibrotic stenosis than the MMH group (41). In the present study, none of the patients developed postoperative anal stenosis after a modified TST procedure. This is associated with conformal selective resections of the rectal mucosa to preserve the mucosal bridges and the normal non-hemorrhoidal-bearing area. Injury of the underlying anal sphincter muscle may also lead to functional alterations (21, 29). Normal rectal compliance was maintained to reduce the risk of anal stenosis, leading to improved anal functional outcomes.

Although the differences in the incidences of anastomotic bleeding, anal discharge and rectovaginal fistula were not significant, modified TST had a lower recurrence rate and lower complication rate than TST (21). It was speculated that modified TST is a precise, conformal selective resection, not a full or partial circumference excision. The tissue between the mucosectomies and the protected tissue adjacent to the rectovaginal septum in women was untouched, leading to minimization of the risk for the development of anal stenosis and RVF formation (38).

It has been shown that modified TST solves the dilemma of choosing only single open, double open or triple open anoscopy in TST operations. In addition, it resects the abnormal tissues more accurately and retains more of the normal mucosa. The surgical process is brief, safe, and inexpensive, with fewer complications and a higher quality of life for the patients. We examined the database to analyze the operation cost of TST, and it was 469.2–625.6 dollars, while modified TST was only 156.4–234.6 dollars. Yang et al. (42) estimated that the overall expenditure on hemorrhoids in the US employer-insured population was $770 million annually. However, Chinese patients generally stay in the hospital longer and are discharged from the hospital with less pain, unobstructed stool and better recovery. The modified TST procedure reduced the financial stress on the patients during their longer hospital stay.

## Conclusions

In summary, modified TST can be used to precisely resect prolapsing hemorrhoids and effectively preserve anal sphincter function and the normal perianal mucosa in patients. The technique is associated with fewer complications and lower recurrence rates. Modified TST is therefore considered a satisfactory and economical surgical procedure for prolapsing hemorrhoids.

## Data Availability Statement

The original contributions presented in the study are included in the article/Supplementary Material, further inquiries can be directed to the corresponding author.

## Ethics Statement

The studies involving human participants were reviewed and approved by the Ethics Committee of the Second Affiliated Hospital and Yuying Children's Hospital of Wenzhou Medical University. The patients/participants provided their written informed consent to participate in this study.

## Author Contributions

CY performed the majority of the data analysis and wrote the article. CZho provided advice on the design and performance of the study. RX performed the follow-up and the initial data analysis. XJ performed ethical supervision and administrative support. CJ performed the majority of the clinical therapy. CZhe provided study materials or patients and is responsible for the article's reliability. All authors contributed to the article and approved the submitted version.

## Funding

This article was supported by the Foundation of Science and Technology Bureau of WenZhou (Y20210938).

## Conflict of Interest

The authors declare that the research was conducted in the absence of any commercial or financial relationships that could be construed as a potential conflict of interest.

## Publisher's Note

All claims expressed in this article are solely those of the authors and do not necessarily represent those of their affiliated organizations, or those of the publisher, the editors and the reviewers. Any product that may be evaluated in this article, or claim that may be made by its manufacturer, is not guaranteed or endorsed by the publisher.
